# Laboratory and Clinical Practices in the Study of Coeliac Disease in Children and Adults: Recommendations from a Spanish Multicentre Survey

**DOI:** 10.3390/nu17122032

**Published:** 2025-06-18

**Authors:** Rocío Aguado, Juan Irure-Ventura, Maria Luisa Vargas, Garbiñe Roy, Yvelise Barrios, Laura Martínez-Martínez, Beatriz Rodríguez, Marco Antonio Montes-Cano, Marcos López-Hoyos, Aurora Jurado

**Affiliations:** 1 UGC Inmunologia-Alergologia, Maimonides Biomedical Research Institute of Cordoba (IMIBIC), Reina Sofia University Hospital of Cordoba, 14004 Cordoba, Spain; rocio.aguado.alvarez.sspa@juntadeandalucia.es (R.A.); aurora.jurado.sspa@juntadeandalucia.es (A.J.); 2 Immunology Department, University Hospital Marqués de Valdecilla, 39008 Santander, Spain; juan.irure@scsalud.es; 3 Immunopathology Group, University Hospital Marqués de Valdecilla-IDIVAL, 39011 Santander, Spain; 4 Immunology and Genetics Department, Hospital Universitario de Badajoz, 06080 Badajoz, Spain; marialuisa.vargas@salud-juntaex.es; 5 Immunology Department, Hospital Universitario Ramon y Cajal, Instituto Ramón y Cajal de Investigación Sanitaria (IRYCIS), 28034 Madrid, Spain; mgroy@med.ucm.es; 6 Immunology Laboratory, Hospital Universitario de Canarias, 38320 Santa Cruz de Tenerife, Spain; yvelise.barrios@gmail.com; 7 Immunology Department, Hospital de La Santa Creu I Sant Pau, Universitat Autonoma de Barcelona, C/Sant Antoni M Claret 167, 08025 Barcelona, Spain; lmartinezma@santpau.cat; 8 Immunology Department, Hospital Universitario Virgen del Rocio (IBiS, CSIC, US), 41013 Seville, Spain; beatriz.rodriguezbayona@gmail.com (B.R.); marcoa.montes.sspa@juntadeandalucia.es (M.A.M.-C.); 9 Department of Molecular Biology, University of Cantabria, 39011 Santander, Spain

**Keywords:** coeliac disease, autoimmune disorders, nutritional deficiencies, clinical laboratory diagnostics, RAND/UCLA method, health care surveys, clinical guidelines

## Abstract

**Background/Objectives:** Coeliac disease is an immune-mediated disorder of the gastrointestinal tract that may result in significant nutritional deficiencies. Effective management requires strict, lifelong adherence to a gluten-free diet. Both underdiagnosis and unnecessary dietary restrictions can adversely affect patients’ health and quality of life. To assess adherence to the current recommendations for the laboratory diagnosis of coeliac disease and promote evidence-based practices while reducing inter-laboratory variability, the Spanish Group on Autoimmunity of the Spanish Society of Immunology conducted a nationwide survey. **Methods:** A thirty-item survey was distributed to fifty autoimmune laboratories across Spain. Data were collected through a structured Excel-based questionnaire comprising multiple-choice items, which was distributed via email to the participating laboratories. It explored practices related to the diagnosis of coeliac disease in the general population and among at-risk groups as well as approaches to patient follow-up and demand management. **Results:** Thirty-five laboratories completed the electronic questionnaire. For the serological screening of coeliac disease, all the respondents reported using IgA anti-tissue transglutaminase (tTG-IgA) antibody testing together with total IgA measurement to assess IgA competence. However, consistent use of anti-endomysial antibody testing and HLA genotyping and adherence to pre-analytical recommendations for accurate interpretation of results were not uniform across centres. **Conclusions:** At the time these data were collected (the third trimester of 2021), the 2020 ESPGHAN guidelines for the diagnosis of coeliac disease in the paediatric population had not yet been fully implemented in most of the laboratories surveyed. For diagnosing adults, most laboratories adhered to local and European guidelines.

## 1. Introduction

Coeliac disease (CD) is an immune-mediated disorder that primarily affects the gastrointestinal tract. If left untreated, it can lead to significant nutritional deficiencies and may progress to a systemic condition. In addition, a diagnosis of coeliac disease has a substantial impact on patients’ quality of life. The mainstay of treatment—adherence to a strict gluten-free diet—requires considerable changes in food choices and eating habits, which can also contribute to nutritional deficiencies [1,2,3,4,5,6].

Although the classical approach to coeliac disease diagnosis relies on clinical assessment, the detection of disease-specific autoantibodies, and histological examination of small intestinal biopsies, current European guidelines support a biopsy-free diagnostic pathway for selected patients based on their autoantibody profiles [7,8]. Moreover, the latest updates to these guidelines further expand and refine the recommendations, promoting an evidence-based diagnostic approach that relies on serological testing [9,10]. Since these guidelines’ seminal publication in 2012, several studies have evaluated adherence to them and assessed their performance in diagnosing coeliac disease both in children [11,12,13,14,15] and adults [16,17,18]. For the diagnosis of coeliac disease in adults, local guidelines (such as the Protocol for the Early Diagnosis of Coeliac Disease published by the Spanish Ministry of Health) and recommendations from other gastroenterology societies (such as the British Society of Gastroenterology and the Italian Society of Gastroenterology) have established the need to conduct an intestinal biopsy to confirm this diagnosis, a requirement that remains essential for adults, in contrast to paediatric guidelines [8,19,20].

The paradigm shift, which assigns substantial diagnostic value to the presence of autoantibodies, has not been equally embraced by clinicians or patients. In fact, recent publications suggest that dietary transgressions and suboptimal disease control are more frequent among paediatric patients diagnosed through the non-biopsy approach than among those diagnosed via intestinal biopsy [21]. Furthermore, adherence to the ESPGHAN recommendations has been inconsistent among physicians responsible for autoantibody assessment.

The Spanish Group on Autoimmunity (GEAI) of the Spanish Society of Immunology holds annual scientific and organisational meetings. At the 2021 meeting, the relevance and timeliness of conducting a survey to analyse standard practices in the immunological laboratory diagnosis of CD, both in paediatric and adult populations, were discussed. The rationale was based on the immune-mediated nature of the disease, its high prevalence, the risk of underdiagnosis, and the considerable workload it imposes on autoimmunity laboratories.

In addition to assessing current practices across different laboratories, the ultimate aim of this survey was to reduce variability by comparing these practices with the current recommendations of European scientific societies [9,10].

## 2. Materials and Methods

### 2.1. Study Design, Settings, and Participants

The method selected for this survey was the RAND-UCLA Appropriateness Method [22], which is based on scientific evidence and the collective judgement of a panel of experts. This method combines elements of both the Delphi technique and the nominal group technique. Once developed, the survey was distributed to 50 Spanish laboratories involved in the immunological diagnosis of coeliac disease. Data were collected between April and July 2021. On 3 November 2021, the results of the survey were evaluated by the GEAI’s Board of Directors, using as references the “Protocol for the Early Diagnosis of Coeliac Disease”, published by the Spanish Ministry of Health, Social Services, and Equality in 2018 [8], and the most recent recommendations for the diagnosis of coeliac disease in the paediatric population, issued by the European Society for Paediatric Gastroenterology, Hepatology, and Nutrition (ESPGHAN) in January 2020 [9]. A structured Excel-based questionnaire with multiple-choice items was distributed via email to all members of the GEAI and the Autoimmunity Group of the Spanish Society of Clinical Chemistry (SEQC), from whom data were subsequently collected.

### 2.2. Survey

The electronic survey consisted of 29 multiple-choice questions. Items were grouped according to their focus into the following categories: (1) diagnosis of coeliac disease in the general population; (2) diagnosis in at-risk groups; (3) patient follow-up; and (4) the appropriateness of demand management practices.

The design of the survey was presented at the GEAI assembly during the 42nd Congress of the Spanish Society of Immunology (SEI). All questions and responses are detailed in Table 1. For clarity, and with a few necessary exceptions, response options that received no answers were omitted from the table.

Alongside the results, the assessments provided by GEAI Board members is included, together with the relevant recommendations from ESPGHAN [9] and the Spanish Ministry of Health, Social Services, and Equality [8], as well as the respondents’ specific comments on certain questions.

### 2.3. Statistical Analysis

Statistical analysis was performed using IBM SPSS Statistics, version 24. For each survey question, consensus was defined as ≥75% of participating laboratories selecting the same response option. Descriptive statistics are reported as absolute and relative frequencies for the various response options for each question.

## 3. Results

**Figure 1:**
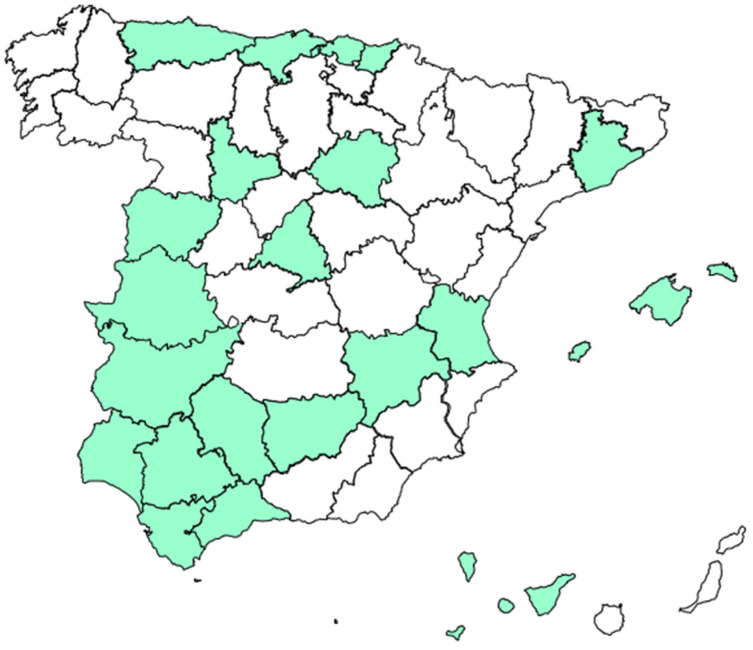
Geographical distribution of participating laboratories.

Thirty-five of the fifty autoimmunity laboratories surveyed (70%), distributed across various regions of Spain, completed the survey (Figure 1).

### 3.1. Diagnostic Questions Regarding Coeliac Disease in the General Population

**Figure 2:**
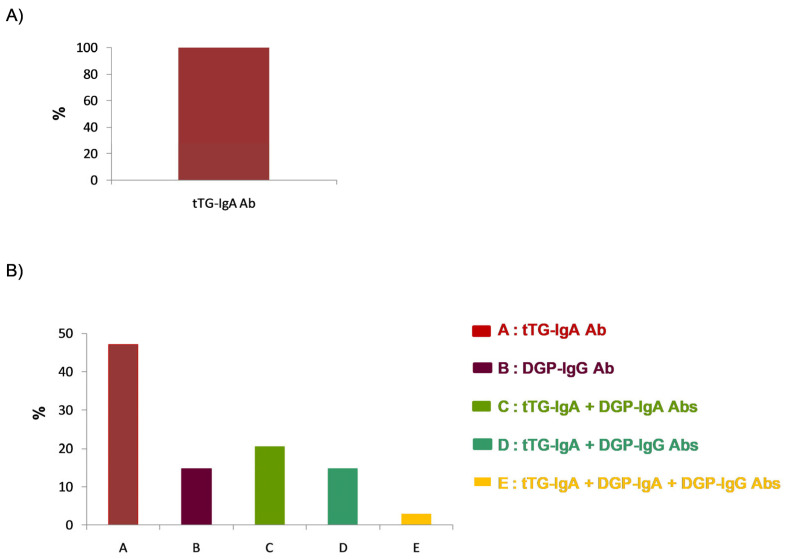
Initial screening strategy for the diagnosis of coeliac disease: autoantibody determination as the initial diagnostic approach for patients (**A**) over 2 years of age and (**B**) under 2 years of age.

Although 77.1% of the respondents reported using different serological approaches depending on patient age, all laboratories used IgA anti-tissue transglutaminase (tTG-IgA) antibodies as a screening method for coeliac disease in symptomatic patients. Similarly, 100% of the respondents used tTG-IgA antibody determination as the initial method for diagnosing coeliac disease in symptomatic patients over two years of age (Figure 2A). However, regarding symptomatic patients under two years of age, 45.7% of the respondents used isolated anti-tTG-IgA antibody testing as the initial diagnostic method. For this patient group, 37.5% of the respondents used anti-tTG-IgA antibodies in combination with anti-deamidated gliadin peptide (DGP) antibodies of the IgA or IgG isotype (DGP-IgA or DGP-IgG), while 14.2% reported using DGP-IgG antibodies alone as the initial screening method (Figure 2B). Regarding the type of gliadin used, 87.5% of the respondents reported not using native gliadin for the determination of anti-gliadin antibodies.

**Figure 3:**
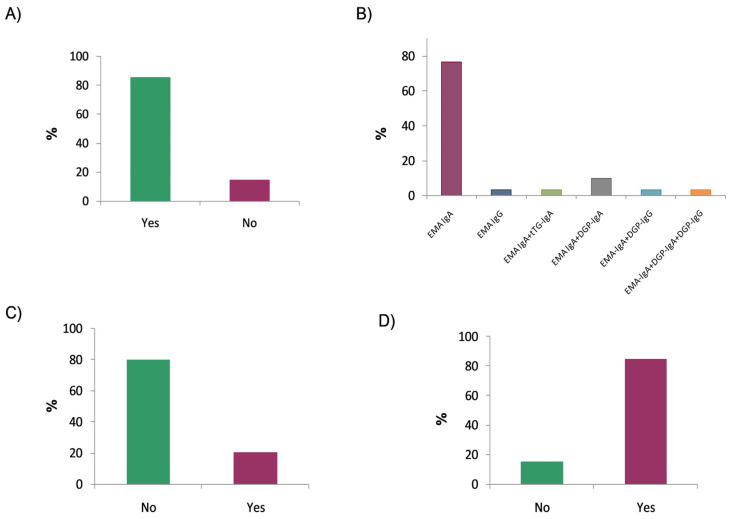
Expanded autoantibody evaluation for patients with positive serological results: (**A**) Extension of initial positive screening with additional autoantibody testing; (**B**) use of anti-endomysial antibodies (EMAs) alone or in combination with other autoantibodies; (**C**) use of a new sample for extended serological testing; and (**D**) use of EMA testing to confirm a positive tTG-IgA result with values <10× the upper limit of normal.

When asked whether they performed additional antibody testing after an initial positive screening, 85.3% of the respondents reported that they did so (Figure 3A). Specifically, in 76.7% of cases, only IgA anti-endomysial antibodies (EMA-IgA) were used, while IgG anti-endomysial antibodies (EMA-IgG) were used in 3.3%. The remaining respondents combined the detection of anti-endomysial antibodies with the determination of other autoantibodies (Figure 3B). Regarding the substrate used for endomysial antibody detection, all laboratories (100%) reported using a distal monkey oesophagus. Approximately half of the laboratories (55.9%) reported EMA results qualitatively, while the remainder used endpoint titration. The survey also inquired whether the extended serological study was performed using the same sample or required a new one; in this case, only 20% of the laboratories reported requiring a new sample (Figure 3C).

Another important aspect concerns whether EMA testing is performed following a positive tTG-IgA screening result that is less than 10 times the upper limit of normal (ULN) established by each laboratory. The survey results showed that 84.8% of the respondents used EMA testing to confirm any positive tTG-IgA result, regardless of the antibody titre (Figure 3D).

Regarding the appropriateness of recommending serological testing at diagnosis while the patient is on a gluten-containing diet, 76.5% of the respondents reported that they do not include such a recommendation in the laboratory report.

When asked about the method used to assess IgA production competence, 80.1% of the respondents reported routinely adding total IgA as a concurrent test. Moreover, 100% indicated using some kind of method to evaluate this parameter.

In 74.3% of the surveyed laboratories, HLA typing was performed in the context of coeliac disease diagnosis. When asked about the possibility of including a comment recommending genetic testing for patients with high clinical suspicion and negative serological results, 91.2% of the respondents reported that they would not include such a comment. Similarly, when asked about recommending genetic testing for patients with high clinical suspicion and strongly positive serological results, 94.3% of the respondents also reported that they would not include any such comment.

Approximately half of the laboratory groups surveyed (52.9%) performed intraepithelial lymphocyte (IEL) testing in the context of coeliac disease diagnosis. Furthermore, 50% of those who did not perform this test considered its implementation feasible within their laboratory portfolio.

### 3.2. Survey Questions Addressing the Diagnosis of Coeliac Disease in At-Risk Groups

A total of 84.4% of the respondents stated that their clinicians screen for coeliac disease in at-risk populations. In these cases, 100% of those who answered affirmatively reported using tTG-IgA antibody determination, either alone or in combination with other autoantibodies. Nevertheless, when asked about the tests used to diagnose patients with selective IgA deficiency, all the respondents (100%) reported using one or more IgG-based serological biomarkers for coeliac disease.

### 3.3. Survey Questions Regarding Follow-Ups for Coeliac Disease Patients

A total of 62.9% of the respondents reported distinguishing between the initial diagnosis and follow-up cases in coeliac disease testing. When asked about the serological biomarkers used for patient follow-ups, 76.5% reported using isolated tTG-IgA antibodies, and 91% used such autoantibodies in combination with others (DGP and EMA).

Finally, when asked about the recommended frequency of conducting analytical follow-ups for coeliac disease patients, 82.9% of the respondents indicated that they did not apply any specific criteria to regulate or recommend the timing of follow-ups.

### 3.4. Questions Addressing Demand Management for Tests

The laboratory groups were asked whether they used any demand management mechanisms for test requests concerning patients with moderate or low clinical suspicion and negative serological results. A total of 82.9% of the respondents reported not using any such mechanism. Among the small percentage (17.1%) of respondents who did implement some form of demand control, there was no majority consensus regarding the appropriate time interval before repeating serological testing.

Additionally, the respondents were asked about the desirability of implementing such measures in general, and 72.4% expressed agreement.

Finally, 52.4% of the respondents who supported demand control measures considered a six-month interval appropriate before repeating serological testing in cases with negative results and low to moderate clinical suspicion.

## 4. Discussion

The diagnosis of coeliac disease in the paediatric population has evolved substantially following the release of the ESPGHAN recommendations. These guidelines have prompted efforts to extend similar diagnostic criteria to adult patients. Despite the advantages of a biopsy-free approach, the adoption and correct interpretation of these guidelines remain inconsistent among attending clinicians, laboratory physicians, and even patients. This limited implementation has repercussions at multiple levels, particularly impacting patient health through the risk of nutritional deficiencies [2,6].

In this paper, we present the results of a national survey on the reporting and interpretation of coeliac disease diagnostic algorithms in current clinical practice. One of the currently preferred methods for structuring the work of expert panels addressing healthcare issues is the RAND/UCLA Appropriateness Method [22]. This method combines scientific evidence with the expert judgement of a panel. The survey results revealed both concordances and discrepancies with the recommendations issued by ESPGHAN.

In the first block of questions, which focused on the diagnosis of coeliac disease in the general population, all the respondents (100%) reported using the tTG-IgA antibody test as the initial screening method. This finding is consistent with results from similar surveys assessing adherence to the 2020 ESPGHAN guidelines [23]. It may seem striking that most of the laboratory groups reported using a different approach depending on whether a patient is under or over two years of age. However, this apparent contradiction was clarified by subsequent responses, in which most of the laboratory groups indicated that they perform IgA anti-tTG antibody testing, either alone or in combination with other autoantibodies, as also reported in the Australasian survey [18]. In this regard, the 2020 ESPGHAN guidelines recommend the same serological diagnostic approach for children, regardless of age [6]. Similarly, the updated recommendations of the American College of Gastroenterology (ACG), the *Protocol for the Early Diagnosis of Coeliac Disease* published by the Spanish Ministry of Health, and the recommendations of the British and Italian Societies of Gastroenterology endorse the use of tTG-IgA antibodies as the preferred sole test for diagnosing coeliac disease in children under two years of age [8,18,19,24].

On the other hand, most laboratory groups reported performing EMA testing using the same serum sample. In this context, the ESPGHAN, ACG, and Spanish, British, and Italian guidelines recommend requesting a new serum sample [8,9,19,20,24]. Upon reviewing the survey results, the GEAI agreed to recommend including a comment requesting a new sample for EMA testing in such cases. Similarly, the vast majority of laboratories reported using EMA testing to confirm positive tTG-IgA antibody results with values below ten times the upper limit of normal. However, the ESPGHAN criteria, as well as the Spanish protocol, do not recommend employing a second autoantibody when the anti-tTG-IgA levels are below this threshold. In such cases, a biopsy is required to establish diagnostic certainty. For more than a quarter of a century since its initial description, it has been well established that tissue transglutaminase is the main antigen recognised by anti-endomysial antibodies [25]. Similarly, the most widely used substrate for the detection of EMA-IgA is a monkey oesophagus. The results of our survey revealed that a significant proportion of laboratories make extensive use of this resource, either in paediatric patients with positive tTG-IgA results below ten times the upper limit of normal or in non-paediatric patients with a positive result. Therefore, strict adherence to the 2020 ESPGHAN guidelines must be emphasised. Moreover, in the individual comments collected through the survey and during the GEAI assembly where the panel results were presented, concerns were raised regarding the inappropriate or unnecessary use of this resource, particularly given that this tissue is derived from an endangered species.

Three-quarters of the laboratory groups surveyed reported that they do not include a comment recommending that serological testing be performed while a patient is on a gluten-containing diet. Following the review of the survey results, the GEAI agreed to recommend including a comment informing clinicians of the importance of performing serological testing while a patient is on a full gluten-containing diet. The growing popularity of gluten-free diets has had a significant impact on the detection and diagnosis of coeliac disease [26]. Many individuals adopt gluten-free diets without receiving a prior medical diagnosis, a behaviour often driven by health trends or perceived benefits. This practice can mask the symptoms of coeliac disease and complicate the diagnostic process, as the elimination of gluten may lead to false-negative results in serological testing. Therefore, it is of great importance to inform clinicians of the need to ensure that patients understand the necessity of maintaining a gluten-containing diet for an accurate diagnosis of coeliac disease. This practice would also help reduce the number of diagnostic tests repeated within a short timeframe due to inconclusive results stemming from inadequate gluten intake.

Concerning genetic testing, appropriately, most laboratories do not recommend such testing in paediatric cases with clearly positive serological results; however, they also tend not to recommend it in cases of high clinical suspicion with negative serological results. According to current guidelines, HLA typing for the DQ2 and DQ8 alleles is recommended in situations where false-negative serological results are possible, such as in patients with low gluten intake, immunosuppressive treatment, or extraintestinal manifestations. Furthermore, the high negative predictive value of HLA typing is particularly noteworthy, as a negative result effectively rules out coeliac disease in the vast majority of cases.

Notable positive aspects include the widespread adoption of flow cytometry for the analysis of intraepithelial lymphocytes, the fact that 100% of laboratories verify IgA production competence, the appropriate non-recommendation of genetic testing in cases with clearly positive serological results, and the minimal use of native gliadin-based assays. In accordance with the current guidelines [9,24,27], all laboratories reported using at least one IgG-class serological marker for patients with selective IgA deficiency.

The prevalence of coeliac disease has increased in recent years and is now estimated to affect approximately 1% of the global population [28]. In Spain, the reported prevalence ranges from 1 in 71 children to 1 in 357 adults. This high prevalence, combined with the undesirable consequences of underdiagnosis, makes serological testing for coeliac disease one of the most frequently requested analyses in autoimmunity laboratories [29]. Moreover, there is still controversy regarding the optimal monitoring strategies, as there is no consensus on how follow-ups should be conducted, and differences in healthcare provision across systems further hinder the establishment of uniform guidelines [28]. The associated workload and cost make it essential to implement measures aimed at rationalising test demand [30,31]. Therefore, the survey in question included several specific questions addressing this issue. Most laboratories did not apply such measures for patients with moderate or low clinical suspicion and negative serological results, although they considered that such measures should be implemented. In reviewing the survey results, the GEAI considered it advisable to introduce demand management mechanisms based on pathophysiological criteria in cases with negative serological results and low to moderate clinical suspicion. Similarly, in cases where a negative result has been obtained, the GEAI deemed it appropriate not to repeat a serology test within three months in such cases, based on both pathophysiological reasoning and the recommended intervals for repeating autoantibody testing for other conditions.

Other issues, such as the applicability of serological criteria to efforts to avoid conducting an intestinal biopsy in the diagnosis of coeliac disease in adults, were not explicitly addressed in the survey. However, they remain highly relevant, as demonstrated by the differing approaches to this topic reported in various studies [16,17,18,30,31,32].

A theoretical limitation of this survey with respect to the extrapolation of its results to other settings could be the differences in the local diagnostic policies for coeliac disease. However, the local guidelines in Spain regarding the diagnosis of paediatric coeliac disease are aligned with the 2020 ESPGHAN recommendations. Similarly, for adult diagnosis, the local recommendations are consistent with those of other relevant societies, such as the British Society of Gastroenterology and the Italian Societies of Gastroenterology.

## 5. Conclusions

At the time this survey was conducted—six months after the publication of the 2020 ESPGHAN guidelines for the diagnosis of coeliac disease in the paediatric population—most of the laboratories surveyed had not yet implemented the main changes introduced in these recommendations, particularly the age-independent diagnostic approach and the updated guidance on the use of genetic testing. Accordingly, the GEAI recommends that participating laboratories

- Update their protocols in accordance with the 2020 ESPGHAN guidelines and the Spanish protocol for the laboratory diagnosis of coeliac disease;
- Include a comment recommending that anti-EMA testing be performed on a new sample when tTG-IgA antibody levels used for screening exceed ten times the upper limit of normal;
- Refrain from performing anti-EMA testing when tTG-IgA antibody levels are below ten times the upper limit of normal, as such testing is not clinically meaningful and the diagnosis of coeliac disease must be confirmed by an intestinal biopsy;
- Include a comment informing clinicians of the importance of performing serological testing while a patient is on a gluten-containing diet;
- Include a comment recommending HLA typing in cases with a risk of false-negative serological results, such as in cases of low gluten intake, immunosuppressive treatment, or the presence of extraintestinal manifestations;
- Encourage the formation of working groups to develop demand control mechanisms aimed at avoiding unnecessary repetition of laboratory tests in cases with low clinical suspicion.

## Figures and Tables

**Table 1:** Survey questions, response options, and results (%).

Questions	Response Options	Results (%)
1. Do you use a different diagnostic approach to coeliac disease depending on the patient’s age?	Yes	77.1
	No	22.9
2. What method do you use for the initial screening or diagnosis of coeliac disease in symptomatic patients?	tTG-IgA	100
3. What method do you use for the initial screening of coeliac disease in symptomatic patients aged ≤ 2 years?	tTG-IgA	45.7
	tTG-IgA + DGP-IgA	20.3
	DGP-IgG	14.2
	tTG-IgA + DGP-IgG	14.3
	tTG-IgA + DGP-IgA + DGP-IgG	2.9
4. What method do you use for the initial screening of coeliac disease in symptomatic patients aged > 2 years?	tTG-IgA	100
5. For the determination of anti-gliadin antibodies, do you use native gliadin antigens?	Yes	6.3
	No	87.5
	Only in certain cases	6.3
6. What substrate do you use for the determination of EMA (anti-endomysial antibodies)?	Monkey distal oesophagus	100
	Umbilical cord	0
7. How do you report anti-endomysial antibody results?	Positive/Negative	55.9
	Final titre	44.1
8. If the screening result is positive, do you complete the study with additional autoantibody tests?	Yes	85.3
	No	14.7
9. If yes, which autoantibodies are included in the extended study?	tTG-IgA + EMA-IgA	3.3
	DGP-IgA + EMA-IgA	10
	DGP-IgG + EMA-IgA	3.3
	EMA-IgA	76.7
	EMA-IgG	3.3
	DGP-IgA + DGP-IgG + EMA-IgA	3.3
10. If yes, which sample type do you use?	New sample requested	10
	With the same sample	80
	With the same sample but a new one is requested	10
11. After a positive tTG-IgA screening test with levels below 10× the upper limit of normal, do you perform the EMA-IgA test?	Yes	84.8
	No	15.2
12. Do you inform the clinician about the importance of conducting coeliac disease screening while the patient is on a normal gluten-containing diet?	Yes	23.5
	No	76.5
13. What method do you use to assess IgA competence in the patient?	A note is added recommending total IgA testing	2.9
	Medical records are reviewed to assess IgA competence	17.1
	A serum total IgA test is routinely ordered alongside screening	80.1
14. What test do you use for patients with selective IgA deficiency?	tTG-IgG	54.3
	DGP-IgG	14.3
	EMA-IgG	5.7
	tTG-IgG + DGP-IgG	20
	DGP-IgG + EMA-IgG	2.9
	tTG-IgG + DGP-IgG + EMA-IgG	2.9
15. Is screening for coeliac disease in at-risk populations routinely performed by your laboratory users?	Yes	15.6
	No	84.4
16. If yes, which test is used for screening at-risk populations?	tTG-IgA	96.3
	tTG-IgA + DGP-IgG	3.7
17. In cases of high clinical suspicion but negative serology, do you include a comment recommending genetic testing?	Yes	8.8
	No	91.2
18. When both clinical suspicion and serological results are positive, do you add a recommendation for HLA typing?	Yes	5.7
	No	94.3
19. In cases of moderate or low clinical suspicion with negative serology, do you manage the test request (e.g., by cancelling)?	Yes	82.9
	No	17.1
20. If yes, what is the minimum interval you require before re-testing for coeliac disease?	1 month	20
	6 months	30
	1 year	10
	Other	40
21. If a demand control mechanism is not currently used, do you consider it should be?	Yes	72.4
	No	27.6
22. If your answer is that test demand should not be restricted, please provide a brief justification:	Open-ended responses have been excluded for clarity	
23. If yes, what interval would you consider appropriate before repeating serological testing?	1 month	9.5
	6 months	57.2
	1 year	23.8
	Other	9.5
24. Do you distinguish between initial diagnosis and follow-up in coeliac disease testing?	Yes	62.9
	No	37.1
25. What tests do you use to monitor coeliac disease?	tTG-IgA	76.5
	tTG-IgG	2.9
	DGP-IgA	2.9
	tTG-IgA + DGP-IgA	5.7
	tTG-IgA + DGP-IgA + EMA-IgA	8.8
	EMA-IgA	2.9
26. How often do you recommend follow-up for patients diagnosed with coeliac disease?	Every month for lifelong	12.1
	I do not include recommendations in this respect	42.9
	At the clinician’s request	42.5
27. Is HLA typing for coeliac disease diagnosis performed in your unit?	Yes	74.3
	No	25.7
28. Is immunophenotyping of intraepithelial lymphocytes (IELs) from intestinal biopsies performed in your unit?	Yes	47.1
	No	52.9
29. If your unit does not perform IEL immunophenotyping, do you consider it feasible to implement?	Yes	50
	No	50

Percentages represent the proportions of laboratories selecting each option. Open-ended responses and narrative comments were collected but are not included in this article. Abbreviations: tTG, anti-tissue transglutaminase antibodies; DGP, anti-deamidated gliadin peptide antibodies; EMAs, anti-endomysial antibodies; IELs, intraepithelial lymphocytes.

## Data Availability

The raw data supporting the conclusions of this article will be made available by the authors on request.
